# [μ-Bis(diphenyl­arsanyl)methane-1:2κ^2^ *As*:*As*’]nona­carbonyl-1κ^3^ *C*,2κ^3^ *C*,3κ^3^ *C*-[(4-methyl­sulfanylphen­yl)diphenyl­phosphane-3κ*P*]-*triangulo*-triruthenium(0)

**DOI:** 10.1107/S1600536810054206

**Published:** 2011-01-15

**Authors:** Omar bin Shawkataly, Imthyaz Ahmed Khan, H. A. Hafiz Malik, Chin Sing Yeap, Hoong-Kun Fun

**Affiliations:** a Chemical Sciences Programme, School of Distance Education, Universiti Sains Malaysia, 11800 USM, Penang, Malaysia; b X-ray Crystallography Unit, School of Physics, Universiti Sains Malaysia, 11800 USM, Penang, Malaysia

## Abstract

In the title *triangulo*-triruthenium compound, [Ru_3_(C_25_H_22_As_2_)(C_19_H_17_PS)(CO)_9_], the bis­(diphenyl­arsanyl)methane ligand bridges an Ru—Ru bond and the monodentate phosphane ligand bonds to the third Ru atom. Both arsine and phosphane ligands are equatorial with respect to the Ru_3_ triangle. In addition, each Ru atom carries one equatorial and two axial terminal carbonyl ligands. The three phosphane-substituted benzene rings make dihedral angles of 57.91 (19), 84.31 (15) and 59.37 (18)° with each other. The dihedral angles between the two benzene rings are 60.9 (2) and 85.40 (18)° for the two diphenyl­arsanyl groups. In the crystal, mol­ecules are linked into a three-dimensional framework by inter­molecular C—H⋯O hydrogen bonds. Weak inter­molecular C—H⋯π inter­actions stabilize the crystal structure.

## Related literature

For general background to *triangulo*-triruthenium derivatives, see: Bruce *et al.* (1985 ▶, 1988*a* ▶,*b* ▶). For related structures, see: Shawkataly *et al.* (1998 ▶, 2004 ▶, 2010*a* ▶,*b* ▶). For the synthesis of Ru_3_(CO)_10_(μ-Ph_2_AsCH_2_AsPh_2_), see: Bruce *et al.* (1983 ▶) and for that of 4-methyl­thio­phenyl­diphenylphosphane, see: Fuhr *et al.* (2002 ▶). For the stability of the temperature controller used in the data collection, see: Cosier & Glazer (1986 ▶).
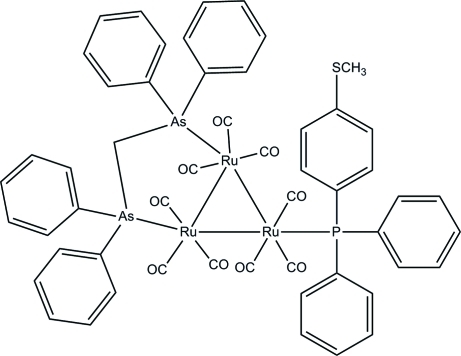


## Experimental

### 

#### Crystal data

[Ru_3_(C_25_H_22_As_2_)(C_19_H_17_PS)(CO)_9_]
*M* *_r_* = 1335.92
Monoclinic, 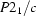

*a* = 16.1070 (11) Å
*b* = 16.7244 (12) Å
*c* = 24.3147 (13) Å
β = 129.712 (3)°
*V* = 5038.6 (6) Å^3^
*Z* = 4
Mo *K*α radiation
μ = 2.32 mm^−1^
*T* = 100 K
0.19 × 0.07 × 0.03 mm

#### Data collection

Bruker APEXII DUO CCD area-detector diffractometer
Absorption correction: multi-scan (*SADABS*; Bruker, 2009 ▶) *T*_min_ = 0.660, *T*_max_ = 0.927
41559 measured reflections
14623 independent reflections
11553 reflections with *I* > 2σ(*I*)
*R*_int_ = 0.036

#### Refinement

*R*[*F* ^2^ > 2σ(*F* ^2^)] = 0.032
*wR*(*F* ^2^) = 0.081
*S* = 1.02
14623 reflections
623 parameters
H-atom parameters constrained
Δρ_max_ = 1.24 e Å^−3^
Δρ_min_ = −0.97 e Å^−3^

### 

Data collection: *APEX2* (Bruker, 2009 ▶); cell refinement: *SAINT* (Bruker, 2009 ▶); data reduction: *SAINT*; program(s) used to solve structure: *SHELXTL* (Sheldrick, 2008 ▶); program(s) used to refine structure: *SHELXTL*; molecular graphics: *SHELXTL*; software used to prepare material for publication: *SHELXTL* and *PLATON* (Spek, 2009 ▶).

## Supplementary Material

Crystal structure: contains datablocks global, I. DOI: 10.1107/S1600536810054206/sj5088sup1.cif

Structure factors: contains datablocks I. DOI: 10.1107/S1600536810054206/sj5088Isup2.hkl

Additional supplementary materials:  crystallographic information; 3D view; checkCIF report

## Figures and Tables

**Table 1:** Hydrogen-bond geometry (Å, °) *Cg*1, *Cg*2 and *Cg*3 are the centroids of the C7–C12, C38–C43 and C14–C19 benzene rings, respectively.

*D*—H⋯*A*	*D*—H	H⋯*A*	*D*⋯*A*	*D*—H⋯*A*
C4—H4*A*⋯O6^i^	0.93	2.48	3.278 (6)	144
C24—H24*A*⋯O7^ii^	0.93	2.56	3.259 (5)	132
C42—H42*A*⋯O1^iii^	0.93	2.57	3.414 (5)	151
C30—H30*A*⋯*Cg*1^iii^	0.93	3.00	3.890 (6)	161
C34—H34*A*⋯*Cg*2^iv^	0.93	2.89	3.785 (4)	163
C40—H40*A*⋯*Cg*3^v^	0.93	2.88	3.672 (4)	144

Symmetry codes: (i) 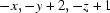; (ii) 
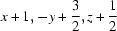
; (iii) 
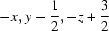
; (iv) 
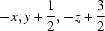
; (v) 
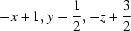
.

**Fig. 1.**
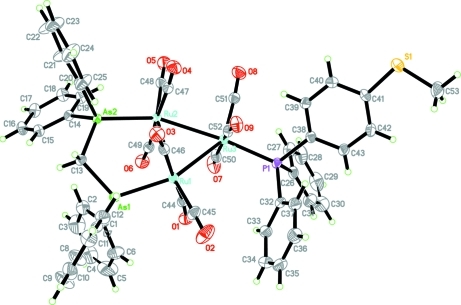
The molecular structure of the title compound with 50% probability ellipsoids for non-H atoms.

**Fig. 2.**
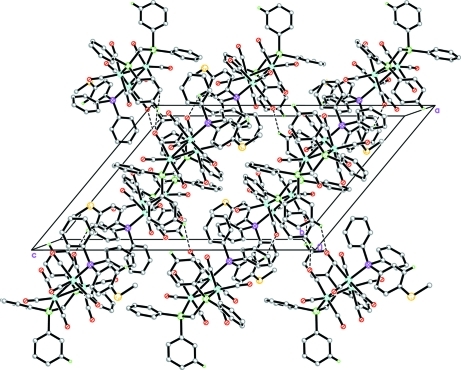
The crystal packing of the title compound, viewed down the b axis, showing the molecules are linked into a three-dimensional framework. Hydrogen atoms not involved in the hydrogen-bonding (dashed lines) have been omitted for clarity.

## supplementary crystallographic information

### Comment

A large number of substituted derivatives of the type Ru_3_(CO)_12-n_*L*_n_ (*L* = group 15 ligand) have been reported (Bruce *et al.*, 1985, 1988*a*,*b*). As part of our study on the substitution of transition metal-carbonyl clusters with mixed-ligand complexes, we have published several structures of *triangulo*-triruthenium-carbonyl clusters containing mixed P/As and P/Sb ligands (Shawkataly *et al.*, 1998, 2004, 2010*a*,*b*). Herein we report the synthesis and structure of the title compound.

The bis(diphenylarsanyl)methane ligand bridges the Ru1—Ru2 bond and the monodentate phosphane ligand bonds to the Ru3 atom. Both arsine and phosphane ligands are equatorial with respect to the Ru_3_ triangle. Additionally, each Ru atom carries one equatorial and two axial terminal carbonyl ligands (Fig 1). The three phosphane-substituted benzene rings make dihedral angles (C26–C31/C32–C37, C26–C31/C38–C43 and C32–C37/C38–C43) of 57.91 (19), 84.31 (15) and 59.37 (18)° with each other respectively. The dihedral angles between the two benzene rings (C1–C6/C7–C12 and C14–C19/C20–C25) are 60.9 (2) and 85.40 (18)° for the two diphenylarsanyl groups respectively. The torsion angle of the methylthio group (C53–S1–C41–C42) being -4.5 (3)°.

In the crystal packing, the molecules are linked into a three-dimensional framework by intermolecular C4—H4A···O6, C24—H24A···O7 and C42—H42A···O1 hydrogen bonds (Fig. 2, Table 1). Weak intermolecular C—H···π interactions (Table 1) stabilize the crystal structure.

### Experimental

All manipulations were performed under a dry oxygen-free nitrogen atmosphere using standard Schlenk techniques. All solvents were dried over sodium and distilled from sodium benzophenone ketyl under dry oxygen free nitrogen. 4-Methylthiophenyldiphenylphosphane (Fuhr *et al.*, 2002) and Ru_3_(CO)_10_(µ-Ph_2_AsCH_2_AsPh_2_) (Bruce *et al.*, 1983) was prepared by reported procedure. The title compound was obtained by refluxing equimolar quantities of Ru_3_(CO)_10_(µ-Ph_2_AsCH_2_AsPh_2_) and 4-methylthiophenyldiphenylphosphane in hexane under nitrogen atmosphere. Crystals suitable for X-ray diffraction were grown by slow solvent / solvent diffusion of CH_3_OH into CH_2_Cl_2_.

### Refinement

All hydrogen atoms were positioned geometrically and refined using a riding model with C—H = 0.93–0.97 Å and *U*_iso_(H) = 1.2 or 1.5 *U*_eq_(C). A rotating group model was applied for the methyl group. The maximum and minimum residual electron density peaks of 1.24 and -0.96 e Å^-3^ were located 0.77 Å and 0.74 Å from the Ru1 and Ru2 atoms, respectively.

### Crystal data

**Table:**

[Ru_3_(C_25_H_22_As_2_)(C_19_H_17_PS)(CO)_9_]	*F*(000) = 2632
*M**_r_* = 1335.92	*D*_x_ = 1.761 Mg m^−^^3^
Monoclinic, *P*2_1_/*c*	Mo *K*α radiation, λ = 0.71073 Å
Hall symbol: -P 2ybc	Cell parameters from 9884 reflections
*a* = 16.1070 (11) Å	θ = 2.8–30.0°
*b* = 16.7244 (12) Å	µ = 2.32 mm^−^^1^
*c* = 24.3147 (13) Å	*T* = 100 K
β = 129.712 (3)°	Needle, brown
*V* = 5038.6 (6) Å^3^	0.19 × 0.07 × 0.03 mm
*Z* = 4	

### Data collection

**Table:**

Bruker APEXII DUO CCD area-detector diffractometer	14623 independent reflections
Radiation source: fine-focus sealed tube	11553 reflections with *I* > 2σ(*I*)
graphite	*R*_int_ = 0.036
φ and ω scans	θ_max_ = 30.0°, θ_min_ = 1.8°
Absorption correction: multi-scan (*SADABS*; Bruker, 2009)	*h* = −22→22
*T*_min_ = 0.660, *T*_max_ = 0.927	*k* = −22→23
41559 measured reflections	*l* = −34→30

### Refinement

**Table:**

Refinement on *F*^2^	Primary atom site location: structure-invariant direct methods
Least-squares matrix: full	Secondary atom site location: difference Fourier map
*R*[*F*^2^ > 2σ(*F*^2^)] = 0.032	Hydrogen site location: inferred from neighbouring sites
*wR*(*F*^2^) = 0.081	H-atom parameters constrained
*S* = 1.02	*w* = 1/[σ^2^(*F*_o_^2^) + (0.043*P*)^2^ + 0.5735*P*] where *P* = (*F*_o_^2^ + 2*F*_c_^2^)/3
14623 reflections	(Δ/σ)_max_ = 0.002
623 parameters	Δρ_max_ = 1.24 e Å^−^^3^
0 restraints	Δρ_min_ = −0.97 e Å^−^^3^

### Special details

**Table:**

Experimental. The crystal was placed in the cold stream of an Oxford Cryosystems Cobra open-flow nitrogen cryostat (Cosier & Glazer, 1986) operating at 100.0 (1) K.
Geometry. All e.s.d.'s (except the e.s.d. in the dihedral angle between two l.s. planes) are estimated using the full covariance matrix. The cell e.s.d.'s are taken into account individually in the estimation of e.s.d.'s in distances, angles and torsion angles; correlations between e.s.d.'s in cell parameters are only used when they are defined by crystal symmetry. An approximate (isotropic) treatment of cell e.s.d.'s is used for estimating e.s.d.'s involving l.s. planes.
Refinement. Refinement of *F*^2^ against ALL reflections. The weighted *R*-factor *wR* and goodness of fit *S* are based on *F*^2^, conventional *R*-factors *R* are based on *F*, with *F* set to zero for negative *F*^2^. The threshold expression of *F*^2^ > σ(*F*^2^) is used only for calculating *R*-factors(gt) *etc*. and is not relevant to the choice of reflections for refinement. *R*-factors based on *F*^2^ are statistically about twice as large as those based on *F*, and *R*- factors based on ALL data will be even larger.

### Fractional atomic coordinates and isotropic or equivalent isotropic displacement parameters (Å^2^)

**Table:**

	*x*	*y*	*z*	*U*_iso_*/*U*_eq_	
Ru1	0.298134 (17)	0.831631 (12)	0.779202 (12)	0.01628 (5)	
Ru2	0.366549 (16)	0.752105 (11)	0.710419 (11)	0.01393 (5)	
Ru3	0.229637 (16)	0.671585 (12)	0.727742 (11)	0.01448 (5)	
As1	0.34320 (2)	0.961468 (15)	0.760642 (15)	0.01670 (6)	
As2	0.48023 (2)	0.861583 (15)	0.726315 (14)	0.01358 (6)	
S1	0.29520 (7)	0.30382 (5)	0.94198 (5)	0.03403 (18)	
P1	0.11515 (5)	0.61875 (4)	0.74696 (4)	0.01794 (13)	
O1	0.06273 (17)	0.85680 (13)	0.64359 (13)	0.0332 (5)	
O2	0.2509 (2)	0.88001 (16)	0.87696 (15)	0.0481 (7)	
O3	0.53465 (17)	0.81202 (12)	0.91484 (12)	0.0290 (5)	
O4	0.55898 (18)	0.66694 (12)	0.84723 (12)	0.0305 (5)	
O5	0.3699 (2)	0.62869 (13)	0.62036 (14)	0.0384 (6)	
O6	0.17484 (18)	0.83165 (13)	0.57129 (12)	0.0314 (5)	
O7	0.03756 (19)	0.70147 (14)	0.56881 (12)	0.0384 (5)	
O8	0.2900 (2)	0.52208 (12)	0.69080 (13)	0.0350 (5)	
O9	0.40236 (17)	0.65945 (12)	0.89112 (11)	0.0274 (4)	
C1	0.2316 (2)	1.01511 (17)	0.67165 (17)	0.0258 (6)	
C2	0.2373 (3)	1.02328 (19)	0.61772 (18)	0.0327 (7)	
H2A	0.2977	1.0049	0.6244	0.039*	
C3	0.1528 (3)	1.0589 (2)	0.5532 (2)	0.0501 (10)	
H3A	0.1564	1.0628	0.5166	0.060*	
C4	0.0652 (3)	1.0880 (3)	0.5430 (2)	0.0597 (12)	
H4A	0.0101	1.1130	0.5002	0.072*	
C5	0.0581 (3)	1.0804 (2)	0.5963 (3)	0.0587 (12)	
H5A	−0.0018	1.1006	0.5893	0.070*	
C6	0.1400 (3)	1.0425 (2)	0.6607 (2)	0.0422 (9)	
H6A	0.1336	1.0355	0.6959	0.051*	
C7	0.3912 (2)	1.04386 (16)	0.83213 (16)	0.0214 (5)	
C8	0.3521 (3)	1.12121 (19)	0.8150 (2)	0.0395 (8)	
H8A	0.3008	1.1363	0.7674	0.047*	
C9	0.3895 (4)	1.1766 (2)	0.8691 (2)	0.0472 (10)	
H9A	0.3613	1.2282	0.8574	0.057*	
C10	0.4683 (3)	1.15544 (19)	0.9401 (2)	0.0368 (8)	
H10A	0.4933	1.1927	0.9761	0.044*	
C11	0.5094 (3)	1.07953 (19)	0.95709 (18)	0.0331 (7)	
H11A	0.5640	1.0655	1.0046	0.040*	
C12	0.4693 (2)	1.02319 (18)	0.90326 (17)	0.0286 (6)	
H12A	0.4955	0.9711	0.9153	0.034*	
C13	0.4691 (2)	0.96050 (15)	0.76505 (15)	0.0188 (5)	
H13A	0.5341	0.9667	0.8144	0.023*	
H13B	0.4647	1.0058	0.7383	0.023*	
C14	0.4548 (2)	0.89586 (15)	0.63982 (14)	0.0168 (5)	
C15	0.4669 (2)	0.97424 (16)	0.62777 (15)	0.0212 (5)	
H15A	0.4911	1.0129	0.6626	0.025*	
C16	0.4429 (2)	0.99524 (17)	0.56330 (16)	0.0257 (6)	
H16A	0.4479	1.0483	0.5543	0.031*	
C17	0.4117 (2)	0.93731 (17)	0.51296 (15)	0.0259 (6)	
H17A	0.3957	0.9513	0.4701	0.031*	
C18	0.4044 (2)	0.85851 (17)	0.52652 (16)	0.0263 (6)	
H18A	0.3868	0.8191	0.4936	0.032*	
C19	0.4231 (2)	0.83779 (16)	0.58862 (15)	0.0214 (5)	
H19A	0.4145	0.7850	0.5962	0.026*	
C20	0.6352 (2)	0.84273 (15)	0.79266 (14)	0.0172 (5)	
C21	0.6878 (3)	0.8239 (2)	0.76698 (18)	0.0362 (8)	
H21A	0.6499	0.8229	0.7179	0.043*	
C22	0.7977 (3)	0.8064 (3)	0.8140 (2)	0.0511 (11)	
H22A	0.8329	0.7937	0.7963	0.061*	
C23	0.8542 (3)	0.8079 (2)	0.8867 (2)	0.0410 (8)	
H23A	0.9271	0.7945	0.9180	0.049*	
C24	0.8029 (3)	0.8291 (2)	0.91276 (18)	0.0397 (8)	
H24A	0.8416	0.8323	0.9618	0.048*	
C25	0.6924 (2)	0.8461 (2)	0.86547 (17)	0.0328 (7)	
H25A	0.6573	0.8597	0.8832	0.039*	
C26	−0.0185 (2)	0.58590 (17)	0.66644 (16)	0.0247 (6)	
C27	−0.0235 (3)	0.54188 (19)	0.61625 (18)	0.0324 (7)	
H27A	0.0397	0.5317	0.6236	0.039*	
C28	−0.1211 (3)	0.5126 (2)	0.5550 (2)	0.0440 (9)	
H28A	−0.1227	0.4827	0.5221	0.053*	
C29	−0.2153 (3)	0.5280 (2)	0.5434 (2)	0.0475 (10)	
H29A	−0.2810	0.5097	0.5020	0.057*	
C30	−0.2115 (3)	0.5704 (2)	0.5933 (2)	0.0500 (10)	
H30A	−0.2749	0.5799	0.5858	0.060*	
C31	−0.1137 (2)	0.5997 (2)	0.6553 (2)	0.0367 (8)	
H31A	−0.1122	0.6282	0.6887	0.044*	
C32	0.0890 (2)	0.68742 (16)	0.79316 (16)	0.0210 (5)	
C33	0.0218 (2)	0.75348 (16)	0.75643 (17)	0.0260 (6)	
H33A	−0.0135	0.7601	0.7080	0.031*	
C34	0.0078 (3)	0.80941 (18)	0.79248 (19)	0.0320 (7)	
H34A	−0.0383	0.8526	0.7678	0.038*	
C35	0.0627 (3)	0.80081 (19)	0.8652 (2)	0.0336 (7)	
H35A	0.0541	0.8388	0.8891	0.040*	
C36	0.1295 (3)	0.73659 (19)	0.90170 (19)	0.0341 (7)	
H36A	0.1657	0.7310	0.9503	0.041*	
C37	0.1433 (2)	0.67957 (17)	0.86618 (17)	0.0268 (6)	
H37A	0.1889	0.6362	0.8913	0.032*	
C38	0.1634 (2)	0.52798 (15)	0.80201 (15)	0.0191 (5)	
C39	0.2553 (2)	0.48814 (16)	0.82327 (15)	0.0209 (5)	
H39A	0.2932	0.5077	0.8091	0.025*	
C40	0.2921 (2)	0.41979 (16)	0.86515 (15)	0.0223 (5)	
H40A	0.3532	0.3937	0.8779	0.027*	
C41	0.2383 (2)	0.38980 (15)	0.88827 (15)	0.0206 (5)	
C42	0.1459 (2)	0.42901 (17)	0.86758 (16)	0.0240 (6)	
H42A	0.1093	0.4102	0.8829	0.029*	
C43	0.1082 (2)	0.49637 (16)	0.82400 (16)	0.0236 (6)	
H43A	0.0450	0.5209	0.8092	0.028*	
C44	0.1501 (2)	0.84081 (16)	0.69206 (17)	0.0242 (6)	
C45	0.2641 (2)	0.86080 (18)	0.83811 (17)	0.0274 (6)	
C46	0.4472 (2)	0.81506 (15)	0.86264 (16)	0.0211 (5)	
C47	0.4857 (2)	0.69993 (16)	0.79802 (16)	0.0215 (5)	
C48	0.3713 (2)	0.67561 (16)	0.65498 (16)	0.0231 (6)	
C49	0.2428 (2)	0.80292 (16)	0.62486 (15)	0.0211 (5)	
C50	0.1099 (2)	0.69369 (17)	0.62778 (16)	0.0246 (6)	
C51	0.2650 (2)	0.57719 (16)	0.70502 (15)	0.0216 (5)	
C52	0.3425 (2)	0.67135 (15)	0.83039 (15)	0.0194 (5)	
C53	0.2174 (3)	0.2842 (2)	0.9688 (2)	0.0429 (9)	
H53A	0.2453	0.2377	0.9990	0.064*	
H53B	0.1436	0.2749	0.9273	0.064*	
H53C	0.2210	0.3293	0.9947	0.064*	

### Atomic displacement parameters (Å^2^)

**Table:**

	*U*^11^	*U*^22^	*U*^33^	*U*^12^	*U*^13^	*U*^23^
Ru1	0.01661 (10)	0.01670 (10)	0.01801 (11)	−0.00152 (7)	0.01220 (9)	−0.00249 (8)
Ru2	0.01341 (9)	0.01460 (9)	0.01386 (10)	−0.00012 (7)	0.00875 (8)	−0.00023 (7)
Ru3	0.01278 (9)	0.01571 (10)	0.01328 (10)	−0.00109 (7)	0.00754 (8)	0.00002 (7)
As1	0.01609 (12)	0.01591 (12)	0.01858 (14)	0.00069 (9)	0.01129 (12)	−0.00089 (10)
As2	0.01239 (12)	0.01535 (12)	0.01197 (12)	0.00032 (9)	0.00731 (10)	0.00076 (9)
S1	0.0378 (4)	0.0299 (4)	0.0409 (5)	0.0113 (3)	0.0281 (4)	0.0160 (3)
P1	0.0141 (3)	0.0199 (3)	0.0190 (3)	0.0006 (2)	0.0102 (3)	0.0025 (3)
O1	0.0187 (11)	0.0406 (13)	0.0310 (13)	0.0015 (9)	0.0116 (10)	−0.0017 (10)
O2	0.0478 (16)	0.0701 (18)	0.0462 (16)	−0.0237 (13)	0.0392 (15)	−0.0314 (14)
O3	0.0225 (11)	0.0317 (11)	0.0220 (11)	0.0001 (9)	0.0092 (10)	0.0004 (9)
O4	0.0251 (11)	0.0290 (11)	0.0265 (12)	0.0067 (9)	0.0113 (10)	0.0087 (9)
O5	0.0584 (17)	0.0286 (12)	0.0450 (15)	−0.0044 (11)	0.0407 (15)	−0.0102 (10)
O6	0.0223 (11)	0.0380 (12)	0.0222 (11)	0.0046 (9)	0.0088 (10)	0.0073 (9)
O7	0.0273 (12)	0.0419 (13)	0.0180 (11)	−0.0101 (10)	0.0014 (10)	0.0039 (10)
O8	0.0475 (15)	0.0246 (11)	0.0422 (15)	0.0024 (10)	0.0329 (13)	−0.0018 (10)
O9	0.0277 (11)	0.0290 (11)	0.0170 (10)	−0.0040 (8)	0.0105 (10)	0.0027 (8)
C1	0.0174 (13)	0.0218 (13)	0.0274 (16)	0.0036 (10)	0.0094 (13)	0.0018 (12)
C2	0.0225 (15)	0.0345 (17)	0.0273 (17)	0.0013 (13)	0.0095 (14)	0.0065 (13)
C3	0.035 (2)	0.056 (2)	0.029 (2)	0.0036 (17)	0.0070 (17)	0.0167 (17)
C4	0.034 (2)	0.056 (3)	0.044 (3)	0.0121 (18)	0.004 (2)	0.023 (2)
C5	0.0236 (18)	0.053 (2)	0.063 (3)	0.0188 (17)	0.011 (2)	0.008 (2)
C6	0.0255 (17)	0.047 (2)	0.049 (2)	0.0115 (15)	0.0211 (18)	0.0067 (17)
C7	0.0244 (14)	0.0220 (13)	0.0240 (14)	−0.0030 (10)	0.0183 (13)	−0.0060 (11)
C8	0.062 (2)	0.0271 (16)	0.0338 (19)	0.0045 (16)	0.033 (2)	−0.0005 (14)
C9	0.086 (3)	0.0215 (16)	0.047 (2)	0.0041 (17)	0.048 (3)	−0.0027 (15)
C10	0.055 (2)	0.0294 (16)	0.039 (2)	−0.0137 (15)	0.036 (2)	−0.0135 (14)
C11	0.0319 (17)	0.0369 (17)	0.0244 (16)	−0.0085 (13)	0.0151 (15)	−0.0105 (13)
C12	0.0245 (15)	0.0258 (15)	0.0299 (17)	−0.0049 (12)	0.0148 (14)	−0.0103 (12)
C13	0.0159 (12)	0.0213 (13)	0.0181 (13)	−0.0016 (10)	0.0104 (11)	−0.0027 (10)
C14	0.0162 (12)	0.0202 (12)	0.0132 (12)	0.0012 (9)	0.0090 (11)	0.0030 (10)
C15	0.0221 (13)	0.0212 (13)	0.0174 (13)	−0.0008 (10)	0.0112 (12)	0.0011 (10)
C16	0.0282 (15)	0.0235 (14)	0.0226 (15)	−0.0015 (11)	0.0150 (13)	0.0051 (11)
C17	0.0263 (15)	0.0326 (15)	0.0142 (13)	−0.0034 (12)	0.0108 (12)	0.0029 (11)
C18	0.0295 (16)	0.0286 (15)	0.0178 (14)	−0.0033 (12)	0.0137 (13)	−0.0027 (12)
C19	0.0259 (14)	0.0176 (12)	0.0195 (14)	−0.0026 (10)	0.0139 (13)	−0.0002 (10)
C20	0.0107 (11)	0.0191 (12)	0.0166 (13)	0.0003 (9)	0.0063 (11)	0.0024 (10)
C21	0.0195 (15)	0.061 (2)	0.0220 (16)	0.0041 (14)	0.0104 (14)	−0.0085 (15)
C22	0.0197 (16)	0.094 (3)	0.034 (2)	0.0078 (18)	0.0144 (16)	−0.014 (2)
C23	0.0148 (14)	0.058 (2)	0.036 (2)	0.0091 (14)	0.0097 (15)	0.0066 (17)
C24	0.0187 (15)	0.070 (2)	0.0204 (16)	0.0041 (15)	0.0077 (14)	0.0133 (16)
C25	0.0183 (14)	0.059 (2)	0.0199 (15)	0.0046 (14)	0.0118 (13)	0.0092 (14)
C26	0.0163 (13)	0.0242 (14)	0.0252 (15)	−0.0027 (10)	0.0093 (12)	0.0055 (11)
C27	0.0253 (15)	0.0383 (17)	0.0255 (16)	−0.0085 (13)	0.0125 (14)	−0.0021 (13)
C28	0.041 (2)	0.045 (2)	0.0294 (19)	−0.0199 (17)	0.0149 (17)	−0.0050 (16)
C29	0.0271 (18)	0.045 (2)	0.034 (2)	−0.0156 (15)	0.0027 (16)	0.0013 (17)
C30	0.0155 (15)	0.046 (2)	0.062 (3)	0.0002 (14)	0.0127 (18)	0.007 (2)
C31	0.0185 (15)	0.0367 (18)	0.045 (2)	−0.0029 (13)	0.0161 (16)	−0.0007 (15)
C32	0.0205 (13)	0.0220 (13)	0.0254 (15)	0.0002 (10)	0.0169 (13)	0.0009 (11)
C33	0.0262 (15)	0.0267 (15)	0.0257 (15)	0.0045 (11)	0.0168 (14)	0.0047 (12)
C34	0.0325 (17)	0.0277 (15)	0.0396 (19)	0.0074 (13)	0.0248 (16)	0.0037 (14)
C35	0.0383 (18)	0.0324 (16)	0.045 (2)	−0.0012 (14)	0.0336 (18)	−0.0054 (15)
C36	0.0415 (19)	0.0359 (17)	0.0312 (18)	−0.0007 (14)	0.0262 (17)	−0.0003 (14)
C37	0.0256 (15)	0.0283 (15)	0.0280 (16)	0.0031 (12)	0.0178 (14)	0.0023 (12)
C38	0.0154 (12)	0.0199 (12)	0.0179 (13)	−0.0025 (10)	0.0088 (11)	0.0005 (10)
C39	0.0222 (13)	0.0217 (13)	0.0210 (14)	0.0008 (10)	0.0147 (12)	0.0015 (11)
C40	0.0212 (13)	0.0234 (13)	0.0213 (14)	0.0022 (10)	0.0131 (12)	0.0000 (11)
C41	0.0225 (13)	0.0163 (12)	0.0179 (13)	−0.0001 (10)	0.0106 (12)	0.0021 (10)
C42	0.0233 (14)	0.0252 (14)	0.0251 (15)	−0.0031 (11)	0.0162 (13)	0.0030 (11)
C43	0.0179 (13)	0.0247 (14)	0.0272 (15)	0.0006 (10)	0.0139 (13)	0.0043 (12)
C44	0.0229 (14)	0.0240 (14)	0.0284 (16)	−0.0032 (11)	0.0177 (14)	−0.0060 (12)
C45	0.0242 (14)	0.0319 (15)	0.0303 (16)	−0.0087 (12)	0.0193 (14)	−0.0101 (13)
C46	0.0264 (14)	0.0171 (12)	0.0229 (14)	−0.0017 (10)	0.0172 (13)	−0.0007 (10)
C47	0.0220 (13)	0.0209 (13)	0.0252 (15)	−0.0018 (11)	0.0167 (13)	−0.0004 (11)
C48	0.0296 (15)	0.0198 (13)	0.0257 (15)	−0.0010 (11)	0.0204 (14)	−0.0005 (11)
C49	0.0175 (13)	0.0234 (13)	0.0203 (14)	−0.0026 (10)	0.0112 (12)	−0.0021 (11)
C50	0.0210 (14)	0.0227 (14)	0.0229 (15)	−0.0045 (11)	0.0107 (13)	0.0013 (11)
C51	0.0192 (13)	0.0235 (13)	0.0204 (14)	−0.0003 (10)	0.0118 (12)	0.0002 (11)
C52	0.0205 (13)	0.0190 (12)	0.0202 (14)	−0.0041 (10)	0.0138 (12)	−0.0015 (10)
C53	0.0381 (19)	0.051 (2)	0.044 (2)	0.0112 (16)	0.0284 (19)	0.0252 (17)

### Geometric parameters (Å, °)

**Table:**

Ru1—C45	1.895 (3)	C13—H13B	0.9700
Ru1—C46	1.932 (3)	C14—C15	1.384 (3)
Ru1—C44	1.934 (3)	C14—C19	1.394 (4)
Ru1—As1	2.4217 (3)	C15—C16	1.398 (4)
Ru1—Ru2	2.8522 (3)	C15—H15A	0.9300
Ru1—Ru3	2.8606 (3)	C16—C17	1.380 (4)
Ru2—C48	1.895 (3)	C16—H16A	0.9300
Ru2—C49	1.930 (3)	C17—C18	1.382 (4)
Ru2—C47	1.938 (3)	C17—H17A	0.9300
Ru2—As2	2.4393 (3)	C18—C19	1.382 (4)
Ru2—Ru3	2.8357 (3)	C18—H18A	0.9300
Ru3—C51	1.878 (3)	C19—H19A	0.9300
Ru3—C52	1.933 (3)	C20—C21	1.372 (4)
Ru3—C50	1.943 (3)	C20—C25	1.379 (4)
Ru3—P1	2.3453 (7)	C21—C22	1.393 (4)
As1—C1	1.936 (3)	C21—H21A	0.9300
As1—C7	1.950 (3)	C22—C23	1.377 (5)
As1—C13	1.962 (2)	C22—H22A	0.9300
As2—C20	1.946 (3)	C23—C24	1.372 (5)
As2—C14	1.954 (2)	C23—H23A	0.9300
As2—C13	1.967 (3)	C24—C25	1.399 (4)
S1—C41	1.756 (3)	C24—H24A	0.9300
S1—C53	1.775 (3)	C25—H25A	0.9300
P1—C32	1.834 (3)	C26—C27	1.383 (4)
P1—C38	1.836 (3)	C26—C31	1.395 (4)
P1—C26	1.839 (3)	C27—C28	1.392 (5)
O1—C44	1.150 (4)	C27—H27A	0.9300
O2—C45	1.140 (3)	C28—C29	1.379 (6)
O3—C46	1.146 (4)	C28—H28A	0.9300
O4—C47	1.151 (3)	C29—C30	1.374 (6)
O5—C48	1.140 (3)	C29—H29A	0.9300
O6—C49	1.142 (3)	C30—C31	1.399 (5)
O7—C50	1.138 (4)	C30—H30A	0.9300
O8—C51	1.144 (3)	C31—H31A	0.9300
O9—C52	1.153 (3)	C32—C37	1.397 (4)
C1—C2	1.378 (4)	C32—C33	1.398 (4)
C1—C6	1.400 (4)	C33—C34	1.395 (4)
C2—C3	1.394 (5)	C33—H33A	0.9300
C2—H2A	0.9300	C34—C35	1.389 (5)
C3—C4	1.358 (6)	C34—H34A	0.9300
C3—H3A	0.9300	C35—C36	1.371 (5)
C4—C5	1.377 (7)	C35—H35A	0.9300
C4—H4A	0.9300	C36—C37	1.397 (4)
C5—C6	1.399 (6)	C36—H36A	0.9300
C5—H5A	0.9300	C37—H37A	0.9300
C6—H6A	0.9300	C38—C39	1.388 (4)
C7—C8	1.381 (4)	C38—C43	1.401 (3)
C7—C12	1.383 (4)	C39—C40	1.387 (4)
C8—C9	1.392 (5)	C39—H39A	0.9300
C8—H8A	0.9300	C40—C41	1.394 (4)
C9—C10	1.385 (6)	C40—H40A	0.9300
C9—H9A	0.9300	C41—C42	1.393 (4)
C10—C11	1.368 (5)	C42—C43	1.392 (4)
C10—H10A	0.9300	C42—H42A	0.9300
C11—C12	1.392 (4)	C43—H43A	0.9300
C11—H11A	0.9300	C53—H53A	0.9600
C12—H12A	0.9300	C53—H53B	0.9600
C13—H13A	0.9700	C53—H53C	0.9600
			
C45—Ru1—C46	90.51 (13)	C15—C14—C19	119.4 (2)
C45—Ru1—C44	93.36 (13)	C15—C14—As2	123.0 (2)
C46—Ru1—C44	175.24 (11)	C19—C14—As2	117.54 (19)
C45—Ru1—As1	100.13 (9)	C14—C15—C16	120.0 (3)
C46—Ru1—As1	90.28 (8)	C14—C15—H15A	120.0
C44—Ru1—As1	91.78 (8)	C16—C15—H15A	120.0
C45—Ru1—Ru2	165.84 (10)	C17—C16—C15	120.1 (3)
C46—Ru1—Ru2	82.08 (8)	C17—C16—H16A	120.0
C44—Ru1—Ru2	93.56 (8)	C15—C16—H16A	120.0
As1—Ru1—Ru2	91.991 (10)	C16—C17—C18	119.8 (3)
C45—Ru1—Ru3	110.76 (9)	C16—C17—H17A	120.1
C46—Ru1—Ru3	101.44 (8)	C18—C17—H17A	120.1
C44—Ru1—Ru3	74.58 (8)	C17—C18—C19	120.5 (3)
As1—Ru1—Ru3	146.643 (11)	C17—C18—H18A	119.7
Ru2—Ru1—Ru3	59.519 (7)	C19—C18—H18A	119.7
C48—Ru2—C49	90.79 (12)	C18—C19—C14	120.1 (2)
C48—Ru2—C47	90.92 (12)	C18—C19—H19A	120.0
C49—Ru2—C47	176.61 (11)	C14—C19—H19A	120.0
C48—Ru2—As2	104.59 (8)	C21—C20—C25	119.6 (3)
C49—Ru2—As2	89.66 (8)	C21—C20—As2	119.9 (2)
C47—Ru2—As2	92.75 (8)	C25—C20—As2	120.5 (2)
C48—Ru2—Ru3	100.01 (8)	C20—C21—C22	120.3 (3)
C49—Ru2—Ru3	90.21 (8)	C20—C21—H21A	119.8
C47—Ru2—Ru3	86.61 (7)	C22—C21—H21A	119.8
As2—Ru2—Ru3	155.395 (11)	C23—C22—C21	120.1 (3)
C48—Ru2—Ru1	159.76 (8)	C23—C22—H22A	120.0
C49—Ru2—Ru1	84.66 (8)	C21—C22—H22A	120.0
C47—Ru2—Ru1	92.74 (8)	C24—C23—C22	119.9 (3)
As2—Ru2—Ru1	95.117 (10)	C24—C23—H23A	120.1
Ru3—Ru2—Ru1	60.388 (8)	C22—C23—H23A	120.1
C51—Ru3—C52	99.57 (12)	C23—C24—C25	119.9 (3)
C51—Ru3—C50	92.14 (12)	C23—C24—H24A	120.0
C52—Ru3—C50	168.17 (12)	C25—C24—H24A	120.0
C51—Ru3—P1	99.62 (8)	C20—C25—C24	120.1 (3)
C52—Ru3—P1	87.27 (8)	C20—C25—H25A	119.9
C50—Ru3—P1	92.44 (8)	C24—C25—H25A	119.9
C51—Ru3—Ru2	86.81 (8)	C27—C26—C31	118.7 (3)
C52—Ru3—Ru2	90.79 (7)	C27—C26—P1	117.8 (2)
C50—Ru3—Ru2	88.19 (8)	C31—C26—P1	123.4 (3)
P1—Ru3—Ru2	173.506 (19)	C26—C27—C28	121.3 (3)
C51—Ru3—Ru1	144.16 (8)	C26—C27—H27A	119.3
C52—Ru3—Ru1	69.84 (8)	C28—C27—H27A	119.3
C50—Ru3—Ru1	99.57 (8)	C29—C28—C27	119.8 (4)
P1—Ru3—Ru1	113.452 (19)	C29—C28—H28A	120.1
Ru2—Ru3—Ru1	60.093 (7)	C27—C28—H28A	120.1
C1—As1—C7	103.01 (12)	C30—C29—C28	119.6 (3)
C1—As1—C13	104.72 (12)	C30—C29—H29A	120.2
C7—As1—C13	98.63 (11)	C28—C29—H29A	120.2
C1—As1—Ru1	116.68 (9)	C29—C30—C31	121.1 (3)
C7—As1—Ru1	117.57 (8)	C29—C30—H30A	119.5
C13—As1—Ru1	113.89 (8)	C31—C30—H30A	119.5
C20—As2—C14	102.46 (11)	C26—C31—C30	119.5 (4)
C20—As2—C13	101.54 (11)	C26—C31—H31A	120.2
C14—As2—C13	104.34 (11)	C30—C31—H31A	120.2
C20—As2—Ru2	116.43 (7)	C37—C32—C33	119.1 (3)
C14—As2—Ru2	115.44 (8)	C37—C32—P1	121.2 (2)
C13—As2—Ru2	114.74 (7)	C33—C32—P1	119.4 (2)
C41—S1—C53	105.61 (15)	C34—C33—C32	119.9 (3)
C32—P1—C38	103.11 (12)	C34—C33—H33A	120.1
C32—P1—C26	105.57 (13)	C32—C33—H33A	120.1
C38—P1—C26	101.02 (12)	C35—C34—C33	120.2 (3)
C32—P1—Ru3	113.81 (9)	C35—C34—H34A	119.9
C38—P1—Ru3	115.89 (8)	C33—C34—H34A	119.9
C26—P1—Ru3	115.78 (9)	C36—C35—C34	120.2 (3)
C2—C1—C6	119.4 (3)	C36—C35—H35A	119.9
C2—C1—As1	122.1 (2)	C34—C35—H35A	119.9
C6—C1—As1	118.5 (3)	C35—C36—C37	120.2 (3)
C1—C2—C3	120.2 (3)	C35—C36—H36A	119.9
C1—C2—H2A	119.9	C37—C36—H36A	119.9
C3—C2—H2A	119.9	C36—C37—C32	120.3 (3)
C4—C3—C2	120.8 (4)	C36—C37—H37A	119.9
C4—C3—H3A	119.6	C32—C37—H37A	119.9
C2—C3—H3A	119.6	C39—C38—C43	117.6 (2)
C3—C4—C5	119.8 (4)	C39—C38—P1	121.46 (19)
C3—C4—H4A	120.1	C43—C38—P1	120.9 (2)
C5—C4—H4A	120.1	C40—C39—C38	121.5 (2)
C4—C5—C6	120.7 (4)	C40—C39—H39A	119.3
C4—C5—H5A	119.7	C38—C39—H39A	119.3
C6—C5—H5A	119.7	C39—C40—C41	120.6 (2)
C5—C6—C1	119.1 (4)	C39—C40—H40A	119.7
C5—C6—H6A	120.5	C41—C40—H40A	119.7
C1—C6—H6A	120.5	C42—C41—C40	118.8 (2)
C8—C7—C12	119.1 (3)	C42—C41—S1	125.7 (2)
C8—C7—As1	123.3 (2)	C40—C41—S1	115.5 (2)
C12—C7—As1	117.6 (2)	C43—C42—C41	120.1 (2)
C7—C8—C9	120.0 (3)	C43—C42—H42A	119.9
C7—C8—H8A	120.0	C41—C42—H42A	119.9
C9—C8—H8A	120.0	C42—C43—C38	121.4 (2)
C10—C9—C8	120.4 (3)	C42—C43—H43A	119.3
C10—C9—H9A	119.8	C38—C43—H43A	119.3
C8—C9—H9A	119.8	O1—C44—Ru1	170.4 (2)
C11—C10—C9	119.7 (3)	O2—C45—Ru1	175.3 (3)
C11—C10—H10A	120.2	O3—C46—Ru1	173.1 (2)
C9—C10—H10A	120.2	O4—C47—Ru2	174.8 (2)
C10—C11—C12	120.0 (3)	O5—C48—Ru2	177.1 (3)
C10—C11—H11A	120.0	O6—C49—Ru2	172.8 (2)
C12—C11—H11A	120.0	O7—C50—Ru3	175.4 (2)
C7—C12—C11	120.7 (3)	O8—C51—Ru3	176.4 (2)
C7—C12—H12A	119.6	O9—C52—Ru3	168.7 (2)
C11—C12—H12A	119.6	S1—C53—H53A	109.5
As1—C13—As2	112.81 (12)	S1—C53—H53B	109.5
As1—C13—H13A	109.0	H53A—C53—H53B	109.5
As2—C13—H13A	109.0	S1—C53—H53C	109.5
As1—C13—H13B	109.0	H53A—C53—H53C	109.5
As2—C13—H13B	109.0	H53B—C53—H53C	109.5
H13A—C13—H13B	107.8		
			
C45—Ru1—Ru2—C48	64.9 (5)	Ru1—As1—C1—C2	−108.3 (2)
C46—Ru1—Ru2—C48	123.9 (3)	C7—As1—C1—C6	−62.1 (3)
C44—Ru1—Ru2—C48	−54.2 (3)	C13—As1—C1—C6	−164.8 (2)
As1—Ru1—Ru2—C48	−146.1 (3)	Ru1—As1—C1—C6	68.2 (3)
Ru3—Ru1—Ru2—C48	15.6 (3)	C6—C1—C2—C3	0.4 (5)
C45—Ru1—Ru2—C49	142.6 (4)	As1—C1—C2—C3	176.9 (3)
C46—Ru1—Ru2—C49	−158.40 (11)	C1—C2—C3—C4	1.8 (6)
C44—Ru1—Ru2—C49	23.51 (12)	C2—C3—C4—C5	−1.8 (7)
As1—Ru1—Ru2—C49	−68.39 (8)	C3—C4—C5—C6	−0.4 (7)
Ru3—Ru1—Ru2—C49	93.29 (8)	C4—C5—C6—C1	2.5 (6)
C45—Ru1—Ru2—C47	−35.2 (4)	C2—C1—C6—C5	−2.5 (5)
C46—Ru1—Ru2—C47	23.78 (11)	As1—C1—C6—C5	−179.2 (3)
C44—Ru1—Ru2—C47	−154.31 (11)	C1—As1—C7—C8	−3.5 (3)
As1—Ru1—Ru2—C47	113.78 (8)	C13—As1—C7—C8	103.9 (3)
Ru3—Ru1—Ru2—C47	−84.53 (8)	Ru1—As1—C7—C8	−133.3 (2)
C45—Ru1—Ru2—As2	−128.2 (4)	C1—As1—C7—C12	177.4 (2)
C46—Ru1—Ru2—As2	−69.22 (8)	C13—As1—C7—C12	−75.2 (2)
C44—Ru1—Ru2—As2	112.68 (8)	Ru1—As1—C7—C12	47.5 (2)
As1—Ru1—Ru2—As2	20.778 (12)	C12—C7—C8—C9	−1.5 (5)
Ru3—Ru1—Ru2—As2	−177.538 (11)	As1—C7—C8—C9	179.4 (3)
C45—Ru1—Ru2—Ru3	49.3 (4)	C7—C8—C9—C10	2.0 (6)
C46—Ru1—Ru2—Ru3	108.32 (8)	C8—C9—C10—C11	−0.2 (6)
C44—Ru1—Ru2—Ru3	−69.78 (8)	C9—C10—C11—C12	−2.1 (5)
As1—Ru1—Ru2—Ru3	−161.683 (12)	C8—C7—C12—C11	−0.8 (4)
C48—Ru2—Ru3—C51	19.80 (13)	As1—C7—C12—C11	178.4 (2)
C49—Ru2—Ru3—C51	110.64 (12)	C10—C11—C12—C7	2.7 (5)
C47—Ru2—Ru3—C51	−70.54 (12)	C1—As1—C13—As2	−93.82 (15)
As2—Ru2—Ru3—C51	−159.73 (9)	C7—As1—C13—As2	160.20 (14)
Ru1—Ru2—Ru3—C51	−165.63 (9)	Ru1—As1—C13—As2	34.79 (15)
C48—Ru2—Ru3—C52	119.35 (12)	C20—As2—C13—As1	−141.11 (13)
C49—Ru2—Ru3—C52	−149.81 (11)	C14—As2—C13—As1	112.66 (14)
C47—Ru2—Ru3—C52	29.01 (11)	Ru2—As2—C13—As1	−14.64 (16)
As2—Ru2—Ru3—C52	−60.18 (8)	C20—As2—C14—C15	−85.2 (2)
Ru1—Ru2—Ru3—C52	−66.08 (8)	C13—As2—C14—C15	20.4 (2)
C48—Ru2—Ru3—C50	−72.44 (13)	Ru2—As2—C14—C15	147.2 (2)
C49—Ru2—Ru3—C50	18.40 (12)	C20—As2—C14—C19	94.7 (2)
C47—Ru2—Ru3—C50	−162.78 (12)	C13—As2—C14—C19	−159.7 (2)
As2—Ru2—Ru3—C50	108.03 (9)	Ru2—As2—C14—C19	−32.9 (2)
Ru1—Ru2—Ru3—C50	102.13 (9)	C19—C14—C15—C16	2.8 (4)
C48—Ru2—Ru3—Ru1	−174.57 (9)	As2—C14—C15—C16	−177.3 (2)
C49—Ru2—Ru3—Ru1	−83.73 (8)	C14—C15—C16—C17	−2.9 (4)
C47—Ru2—Ru3—Ru1	95.09 (8)	C15—C16—C17—C18	0.0 (5)
As2—Ru2—Ru3—Ru1	5.90 (3)	C16—C17—C18—C19	3.1 (5)
C45—Ru1—Ru3—C51	−143.52 (18)	C17—C18—C19—C14	−3.2 (5)
C46—Ru1—Ru3—C51	−48.56 (17)	C15—C14—C19—C18	0.2 (4)
C44—Ru1—Ru3—C51	128.75 (17)	As2—C14—C19—C18	−179.7 (2)
As1—Ru1—Ru3—C51	59.88 (15)	C14—As2—C20—C21	−25.6 (3)
Ru2—Ru1—Ru3—C51	25.04 (15)	C13—As2—C20—C21	−133.3 (2)
C45—Ru1—Ru3—C52	−65.38 (13)	Ru2—As2—C20—C21	101.4 (2)
C46—Ru1—Ru3—C52	29.57 (11)	C14—As2—C20—C25	156.1 (2)
C44—Ru1—Ru3—C52	−153.11 (12)	C13—As2—C20—C25	48.4 (3)
As1—Ru1—Ru3—C52	138.01 (8)	Ru2—As2—C20—C25	−76.9 (2)
Ru2—Ru1—Ru3—C52	103.18 (8)	C25—C20—C21—C22	1.6 (5)
C45—Ru1—Ru3—C50	109.14 (14)	As2—C20—C21—C22	−176.8 (3)
C46—Ru1—Ru3—C50	−155.90 (12)	C20—C21—C22—C23	0.0 (6)
C44—Ru1—Ru3—C50	21.41 (12)	C21—C22—C23—C24	−2.1 (6)
As1—Ru1—Ru3—C50	−47.46 (9)	C22—C23—C24—C25	2.6 (6)
Ru2—Ru1—Ru3—C50	−82.30 (9)	C21—C20—C25—C24	−1.0 (5)
C45—Ru1—Ru3—P1	12.24 (11)	As2—C20—C25—C24	177.3 (3)
C46—Ru1—Ru3—P1	107.19 (8)	C23—C24—C25—C20	−1.0 (5)
C44—Ru1—Ru3—P1	−75.49 (9)	C32—P1—C26—C27	171.3 (2)
As1—Ru1—Ru3—P1	−144.37 (3)	C38—P1—C26—C27	−81.6 (2)
Ru2—Ru1—Ru3—P1	−179.20 (2)	Ru3—P1—C26—C27	44.4 (3)
C45—Ru1—Ru3—Ru2	−168.56 (11)	C32—P1—C26—C31	−11.9 (3)
C46—Ru1—Ru3—Ru2	−73.61 (8)	C38—P1—C26—C31	95.2 (3)
C44—Ru1—Ru3—Ru2	103.71 (8)	Ru3—P1—C26—C31	−138.8 (2)
As1—Ru1—Ru3—Ru2	34.83 (2)	C31—C26—C27—C28	0.9 (5)
C45—Ru1—As1—C1	−98.91 (14)	P1—C26—C27—C28	177.9 (3)
C46—Ru1—As1—C1	170.52 (12)	C26—C27—C28—C29	0.5 (5)
C44—Ru1—As1—C1	−5.19 (13)	C27—C28—C29—C30	−1.6 (6)
Ru2—Ru1—As1—C1	88.44 (10)	C28—C29—C30—C31	1.2 (6)
Ru3—Ru1—As1—C1	58.93 (10)	C27—C26—C31—C30	−1.3 (5)
C45—Ru1—As1—C7	24.23 (14)	P1—C26—C31—C30	−178.1 (3)
C46—Ru1—As1—C7	−66.34 (12)	C29—C30—C31—C26	0.3 (6)
C44—Ru1—As1—C7	117.95 (12)	C38—P1—C32—C37	25.6 (3)
Ru2—Ru1—As1—C7	−148.42 (9)	C26—P1—C32—C37	131.2 (2)
Ru3—Ru1—As1—C7	−177.93 (9)	Ru3—P1—C32—C37	−100.8 (2)
C45—Ru1—As1—C13	138.84 (13)	C38—P1—C32—C33	−160.9 (2)
C46—Ru1—As1—C13	48.28 (12)	C26—P1—C32—C33	−55.3 (2)
C44—Ru1—As1—C13	−127.43 (12)	Ru3—P1—C32—C33	72.7 (2)
Ru2—Ru1—As1—C13	−33.81 (9)	C37—C32—C33—C34	−1.5 (4)
Ru3—Ru1—As1—C13	−63.32 (9)	P1—C32—C33—C34	−175.1 (2)
C48—Ru2—As2—C20	−73.55 (13)	C32—C33—C34—C35	1.7 (5)
C49—Ru2—As2—C20	−164.28 (12)	C33—C34—C35—C36	−1.1 (5)
C47—Ru2—As2—C20	18.12 (12)	C34—C35—C36—C37	0.3 (5)
Ru3—Ru2—As2—C20	105.97 (9)	C35—C36—C37—C32	−0.2 (5)
Ru1—Ru2—As2—C20	111.12 (9)	C33—C32—C37—C36	0.8 (4)
C48—Ru2—As2—C14	46.67 (13)	P1—C32—C37—C36	174.3 (2)
C49—Ru2—As2—C14	−44.06 (11)	C32—P1—C38—C39	−132.6 (2)
C47—Ru2—As2—C14	138.34 (11)	C26—P1—C38—C39	118.4 (2)
Ru3—Ru2—As2—C14	−133.81 (8)	Ru3—P1—C38—C39	−7.6 (3)
Ru1—Ru2—As2—C14	−128.66 (8)	C32—P1—C38—C43	47.5 (3)
C48—Ru2—As2—C13	168.08 (13)	C26—P1—C38—C43	−61.5 (3)
C49—Ru2—As2—C13	77.35 (12)	Ru3—P1—C38—C43	172.5 (2)
C47—Ru2—As2—C13	−100.25 (12)	C43—C38—C39—C40	−0.4 (4)
Ru3—Ru2—As2—C13	−12.39 (10)	P1—C38—C39—C40	179.7 (2)
Ru1—Ru2—As2—C13	−7.25 (9)	C38—C39—C40—C41	−1.1 (4)
C51—Ru3—P1—C32	164.97 (14)	C39—C40—C41—C42	1.0 (4)
C52—Ru3—P1—C32	65.71 (13)	C39—C40—C41—S1	−179.1 (2)
C50—Ru3—P1—C32	−102.45 (14)	C53—S1—C41—C42	−4.5 (3)
Ru1—Ru3—P1—C32	−0.92 (11)	C53—S1—C41—C40	175.6 (2)
C51—Ru3—P1—C38	45.64 (13)	C40—C41—C42—C43	0.7 (4)
C52—Ru3—P1—C38	−53.62 (13)	S1—C41—C42—C43	−179.2 (2)
C50—Ru3—P1—C38	138.22 (13)	C41—C42—C43—C38	−2.3 (5)
Ru1—Ru3—P1—C38	−120.25 (10)	C39—C38—C43—C42	2.1 (4)
C51—Ru3—P1—C26	−72.42 (14)	P1—C38—C43—C42	−178.0 (2)
C52—Ru3—P1—C26	−171.68 (13)	C51—Ru3—C52—O9	−60.8 (12)
C50—Ru3—P1—C26	20.16 (13)	C50—Ru3—C52—O9	127.4 (11)
Ru1—Ru3—P1—C26	121.69 (10)	P1—Ru3—C52—O9	38.5 (12)
C7—As1—C1—C2	121.3 (3)	Ru2—Ru3—C52—O9	−147.7 (12)
C13—As1—C1—C2	18.6 (3)	Ru1—Ru3—C52—O9	154.8 (12)

### Hydrogen-bond geometry (Å, °)

**Table:**

Cg1, Cg2 and Cg3 are the centroids of the C7–C12, C38–C43 and C14–C19 benzene rings, respectively.

**Table:**

*D*—H···*A*	*D*—H	H···*A*	*D*···*A*	*D*—H···*A*
C4—H4A···O6^i^	0.93	2.48	3.278 (6)	144
C24—H24A···O7^ii^	0.93	2.56	3.259 (5)	132
C42—H42A···O1^iii^	0.93	2.57	3.414 (5)	151
C30—H30A···Cg1^iii^	0.93	3.00	3.890 (6)	161
C34—H34A···Cg2^iv^	0.93	2.89	3.785 (4)	163
C40—H40A···Cg3^v^	0.93	2.88	3.672 (4)	144

Symmetry codes: (i) −*x*, −*y*+2, −*z*+1; (ii) *x*+1, −*y*+3/2, *z*+1/2; (iii) −*x*, *y*−1/2, −*z*+3/2; (iv) −*x*, *y*+1/2, −*z*+3/2; (v) −*x*+1, *y*−1/2, −*z*+3/2.
